# Association of overweight with postoperative acute kidney injury among patients receiving orthotopic liver transplantation: an observational cohort study

**DOI:** 10.1186/s12882-020-01871-0

**Published:** 2020-06-11

**Authors:** Jian Zhou, Lin Lyu, Lin Zhu, Yongxin Liang, He Dong, Haichen Chu

**Affiliations:** 1 Department of Anesthesiology, Qingdao University Medical College, Qingdao, China; 2 Department of Anesthesiology, The Affiliated Hospital of Qingdao University, No. 59, Haier Road, Qingdao, 266100 Shandong Province China

**Keywords:** Overweight, Body mass index, BMI, Obesity paradox, Acute kidney injury, AKI, Orthotopic liver transplantation

## Abstract

**Background:**

Acute kidney injury (AKI) is a common postoperative complication of orthotopic liver transplantation (OLT). So far, little attention has been paid on the association between overweight and AKI after OLT, and animal models or clinical studies have drawn conflicting conclusions. The objective of our study was to determine whether overweight (BMI [Body Mass Index] ≥ 25 kg/m^2^) is associated with an increased risk of AKI after OLT.

**Methods:**

This retrospective cohort study included 244 patients receiving OLT in the Affiliated Hospital of Qingdao University between January 1, 2017, and August 29, 2019. Preoperative, intraoperative, and postoperative data were collected retrospectively. The primary outcome was the development of AKI as defined by Kidney Disease, Improving Global Outcome (KIDGO) staging system. Logistic regression analysis was used to determine the relationship between overweight and the occurrence of postoperative AKI. Data analysis was conducted from September to October 2019, revision in April 2020.

**Results:**

Among 244 patients receiving OLT (mean [standard deviation] age, 54.1 [9.6] years; 84.0% male) identified, 163 patients (66.8%) developed postoperative AKI. Overweight (BMI ≥ 25 kg/m^2^) was associated with a higher rate of postoperative severe AKI (stage 2/3) compared with normal weight (18.5 ≤ BMI < 25 kg/m^2^) (41 [47.7%] vs 39 [28.7%]; adjusted odds ratio [OR], 2.539; 95% confidence interval [CI], 1.389–4.642; *P* = 0.002). Furthermore, patients with obese were at even higher risk of postoperative severe AKI after controlling for confounding factors (adjusted OR: 3.705; 95% CI: 1.108–12.388; *P* = 0.033).

**Conclusions:**

Overweight is independently associated with an increased risk of postoperative severe AKI among patients receiving OLT. The association of BMI with severe AKI after OLT is J-shaped.

## Background

Acute kidney injury (AKI) is a common postoperative complication of orthotopic liver transplantation (OLT), with varying occurrence ranging from 5 to 94% [1]. This extreme variability may be due to the absence of a uniform definition of AKI and rapid improvement of surgical technique and perioperative management over the past few decades. Alternatively, the study subjects are diverse in this setting. Among patients who develop AKI after OLT, approximately 8–17% need renal replacement therapy (RRT) [2]. AKI after OLT was associated with immediate complications including volume overload, metabolic acidosis, and electrolyte disturbances [3] and an increased rate of inferior long-term outcomes such as mortality, graft loss, infection, chronic kidney disease, prolonged stay in the intensive care unit (ICU), and augmented hospital costs [1, 4].

A wide variety of mechanisms have been reported for the development of AKI after OLT, such as female gender, pre-existing diabetes mellitus (DM) [5], the severity of liver disease [6], preoperative renal dysfunction [7–9], perioperative events especially hepatic ischaemia reperfusion injury (IRI) [10, 11], toxicity of immunosuppressive therapy [2, 12, 13], and other miscellaneous etiologies. As the etiology of AKI after OLT are multifactorial and not well understood, timely preventive therapy or medical interventions performed during the initiation phase of AKI can minimize the extent of injury and promote renal recovery, therefore the cornerstone for reducing the development of AKI after OLT is early recognition of high-risk patients alongside active perioperative management.

Generally, overweight is exposed to numerous comorbidities and a higher risk of postoperative complications in patients receiving operation procedure especially OLT [14]. However, there is well-grounded body of work demonstrating that patients with overweight actually do better in the long term when compared with normal weight individuals in general surgery [15], and there is anecdotal evidence that patients with overweight might have a better physiological reserve to handle the stress of surgery in clinical practice. So far, little attention has been paid on the association between overweight and AKI after OLT, and animal models or clinical studies have drawn conflicting conclusions [16, 17].

The objective of our study was to determine the association between overweight (BMI [Body Mass Index] ≥ 25 kg/m^2^) and postoperative AKI in patients receiving OLT. Our secondary objective was to assess whether overweight was associated with other postoperative outcomes in patients receiving OLT.

## Methods

### Patient population and data source

This single-center retrospective observational study included all consecutive patients undergoing OLT from January 1, 2017, to August 29, 2019, in the Affiliated Hospital of Qingdao University, Qingdao, China. All surgical procedures were performed by dedicated surgical team with extensive experience in the field. We used electronic health records to extract all study variables and archived paper medical records to identify and correct the outlying values. Data retrieval was performed by 2 reviewers (Yongxin Liang and He Dong) who were blinded to data analysis and data accuracy was verified by reabstracting 10% (*n* = 25) of the study sample. Patients with important missing values (ie, serum creatinine) were excluded from the model. Patients were excluded if they had: (1) Patients under 18 years of age (*N* = 12); (2) Combined transplantation (*N* = 11); (3) RRT before OLT (*N* = 3); (4) kidney transplantation before OLT (*N* = 4); (5) re-transplantation (*N* = 7); and (6) Lack of important data (*N* = 60). Ultimately, 244 patients were enrolled in final analyses.

The study protocol was approved by the Institutional Review Board of the Ethics Committee of the Affiliated Hospital of Qingdao University with waiver of participant consent (protocol number: QYFY WZLL 25725). This study was also performed in accordance with the Declaration of Helsinki (2013) of the World Medical Association [18]. This study followed the Strengthening the Reporting of Observational Studies in Epidemiology (STROBE) reporting guideline [19].

### Primary exposure

Overweight and obesity are defined by the World Health Organization as abnormal or excessive fat accumulation that presents a risk to health [20]. We use the body mass index (BMI), the weight (in kilograms) of a person divided by the square of his or her height (in metres), as a crude population measure of overweight. A person with a BMI equal to or more than 25 is considered overweight. A person with a BMI of 30 or more is generally considered obese. Data for BMI were not adjusted for ascites because the volume of ascites drained at the time of transplantation did not differ significantly between groups.

### Primary outcome

Our primary outcome was the occurrence of AKI after OLT. According to Kidney Disease: Improving Global Outcomes (KDIGO) serum creatinine criteria [21], the AKI after OLT was defined by an increase in serum creatinine (SCr) of 0.3 mg/dL or greater within 48 h or an increase in SCr of 1.5 or greater times baseline (to convert creatinine level to micromoles per liter, multiply by 88.4) within 7 days or a urine volume less than 0.5 ml/kg/hour for 6 h. Baseline SCr measurements were defined as the most recent value of SCr before surgery. We did not use urine volume as a criterion because this was not available for all patients.

### Additional characteristics

We also collected the demographic data and clinical features that might related to AKI of all enrolled patients. These variables included age, gender, personal history of smoking and alcoholism, assessment of comorbidities (hypertension, DM, chronic kidney disease [CKD], hepatitis B virus [HBV] infection, hepatitis C virus [HCV] infection, encephalopathy, and ascites), underlying liver diseases (hepatocellular carcinoma combined with viral hepatitis, hepatocellular carcinoma, viral hepatitis, alcohol-related liver disease and others [Budd Chiari syndrome, Wilson disease, autoimmune hepatitis, drug-induced hepatitis and cryptogenic hepatitis]), Child-Pugh score, Model for End-Stage Liver Disease (MELD) score, preoperative laboratory data (creatinine, sodium, lymphocyte, lactic acid, glucose, albumin, total bilirubin), intraoperative clinical data (estimated blood loss, received albumin, received packed red blood cell [RBC], total volume of transfusion, nadir mean blood pressure, requirement of vasopressors, anhepatic time, cold ischaemia time and duration of surgery), postoperative clinical outcomes (mechanical ventilation, RRT, hospital days and hospital mortality), peak serum aspartate transaminase (AST) within the first 24 h after OLT was used as a measure of IRI [3].

### Statistical analysis

Categorical data were presented as number (percentage) and compared between two groups using the chi-square test or Fisher exact test, as appropriate. Quantitative data were tested for normality by the Kolmogorov-Smirnov test, and data were considered normally distributed if the *P* value was > 0.05. Normally distributed quantitative data were presented as mean ± standard deviation (SD) and compared by the independent-sample t test or ANOVA as appropriate, while nonnormally distributed quantitative data were expressed as median (interquartile range [IQR]) and compared by the Mann-Whitney U test or Kruskal-Wallis H test as appropriate. We stratified baseline demographics and clinical characteristics by AKI episode (yes/no) in order to select potential confounding factors to be included in multivariate analysis. We used multivariable logistic regression to determine the relationship between BMI and AKI after OLT with adjustment for covariates. Patients were assigned to one of four categories: underweight (BMI < 18.5), normal weight (18.5 ≤ BMI < 25), overweight (25 ≤ BMI < 30), or obese (BMI ≥ 30). Non-linear relationships were explored through a restricted cubic spline with four konts at the 5th, 35th, 65th, and 95th centiles. Three models were used, with model 1 being unadjusted; model 2 adjusting model 1 for a priori defined variables based on literature research and clinical relevance (female gender, CTP score, MELD score, pre-existing CKD, pre-existing DM, preoperative SCr, requirement of vasopressors, postoperative peak AST, Tacrolimus); model 3 adjusting model 2 for potential confounding factors based on univariate analyses (female gender, preoperative SCr, preoperative lactic acid, intraoperative RBC transfusion). All regression model results were reported as an odds ratio (OR) with an associated 95% confidence interval (CI). All analyses were performed using SPSS software (SPSS, version 23.0, IBM Corp., Chicago, IL, United States) and STATA software (STATA, version 12.0, Stata Corp LP, College Station, TX, United States).

## Results

### Study population characteristics

**Fig. 1:**
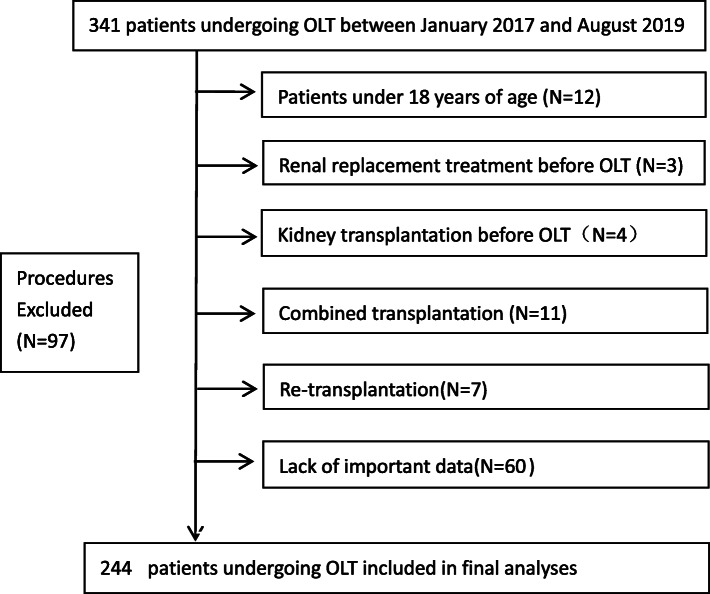
Flow Diagram of Selection of Patients for Inclusion

A total of 244 patients aged 18 years and older receiving OLT were included in the study (Fig. 1). The mean (SD) age was 54.1 (9.6) years, with 205 (84.0%) male and 39 (16.0%) female. At the time of surgery, 88 (36.1%) patients smoked tobacco and 94 (38.2%) had an alcohol consumption. The most common underlying liver disease was hepatocellular carcinoma combined with viral hepatitis (103 [42.2%]) followed by the virus-related hepatic cirrhosis (51 [20.9%]). In total, 163 (66.8%) patients developed postoperative AKI. Compared with patients who did not develop postoperative AKI, these patients had higher BMI (24.69 [22.49–27.12] vs 23.03 [20.81–25.55], *P* = 0.004), preoperative SCr (65.00 [50.00–78.00] vs 71.00 [64.00–87.50], *P* < 0.001) and intraoperative RBC transfusion (12.00 [6.00–14.00] vs 9.00 [4.00–13.50], *P* = 0.026), and a slightly higher proportion of female gender (31 [19.0] vs 8 [9.9], *P* = 0.066) and preoperative blood lactic acid (1.20 [1.00–1.60] vs 1.30 [1.10–1.75], *P* = 0.092), though not statistically significant (0.05 < *P* < 0.10). All demographic and clinical characteristics of the study patients are provided in Table 1.

**Table 1:** Demographic and Clinical Characteristics of the 246 patients, Stratified by AKI

Cohort Characteristics	AKI, No. (%)		*P* Value
	Yes (*n* = 163)	No (*n* = 81)	
Age, mean (SD), y	54.8 (9.6)	53.2 (9.7)	0.281
Female, No. (%)	31 (19.0)	8 (9.9)	0.066
BMI, median (IQR), kg/m^2^	24.7 (22.5–27.1)	23.0 (20.8–25.6)	0.004
Personal history			
Smoking, No. (%)	59 (37.1)	29 (37.7)	0.934
Alcoholism, No. (%)	59 (37.6)	35 (46.1)	0.216
Comorbidities			
Hypertension, No. (%)	44 (27.0)	25 (30.9)	0.527
Diabetes mellitus, No. (%)	58 (35.6)	28 (34.6)	0.876
Chronic kidney disease, No. (%)	20 (12.3)	10 (12.3)	0.986
HBV, No. (%)	78 (47.9)	47 (58.0)	0.134
HCV, No. (%)	6 (3.7)	4 (4.9)	0.641
Encephalopathy, No. (%)	11 (6.7)	6 (7.4)	0.849
Ascites, No. (%)	37 (22.7)	18 (22.2)	0.933
Underlying liver disease			0.356
Hepatocellular carcinoma +Viral hepatitis, No. (%)	65 (39.9)	38 (46.9)	
Hepatocellular carcinoma, No. (%)	36 (22.1)	12 (14.8)	
Viral hepatitis, No. (%)	32 (19.6)	19 (23.5)	
Alcohol-related liver disease, No. (%)	11 (6.7)	7 (8.6)	
Other, No. (%)	19 (11.7)	5 (6.2)	
GFR, median (IQR)	145.0 (108.4–186.3)	134.5 (104.0–164.3)	0.144
Child-Pugh score, median (IQR)	8.0 (7.0–9.0)	8.0 (7.0–9.0)	0.268
MELD score, median (IQR)	6.7 (0.5–12.5)	7.2 (2.1–14.6)	0.474
Preoperative laboratory data			
Creatinine, median (IQR), mg/dL	65.0 (50.0–78.0)	71.0 (64.0–88.0)	< 0.001
Sodium, median (IQR), mEq/L	140.0 (138.0–142.0)	140.0 (137.0–142.0)	0.865
Albumin, median (IQR), g/dL	35.0 (30.6–39.7)	35.3 (30.9–40.6)	0.520
Total bilirubin, median (IQR), umol/L	49.1 (26.6–129.3)	44.6 (20.9–397.0)	0.568
Glucose, median (IQR), mg/dL	5.0 (4.4–6.4)	5.2 (4.6–6.2)	0.282
Lactic acid, median (IQR), mmol/L	1.2 (1.0–1.6)	1.3 (1.1–1.8)	0.092
Surgery			
Estimated blood loss, median (IQR), L	2.0 (1.0–2.5)	1.5 (0.8–2.1)	0.146
RBC transfusion, median (IQR), units	12.0 (6.0–14.0)	9.0 (4.0–13.5)	0.026
Nadir MAP, median (IQR), mmHg	64.9 (61.7–68.5)	64.9 (63.3–69.3)	0.923
Requirement of vasopressors (large dose), No. (%)			
Duration of surgery, median (IQR), min	531.0 (468.0–572.0)	531.0 (470.0–570.0)	0.811
Cold ischemia time, median (IQR), min	382.0 (326.0–384.0)	383.0 (325.0–387.0)	0.628
Anhepatic phase, median (IQR), min	60.0 (52.0–60.0)	60.0 (56.0–64.0)	0.128
Postoperative parameter			
Peak AST, median (IQR), U/L	526.0 (72.5–795.5)	285.0 (79.0–742.0)	0.805
Tacrolimus, median (IQR), ng/mL	9.00 (6.65–11.50)	8.30 (6.30–12.10)	0.657
Postoperative hospitalization, median (IQR), day	27.0 (21.0–30.0)	25.0 (20.0–28.0)	0.334
Hospital mortality, No. (%)	5 (3.1)	2 (2.5)	1.000

### Incidence of AKI

**Fig. 2:**
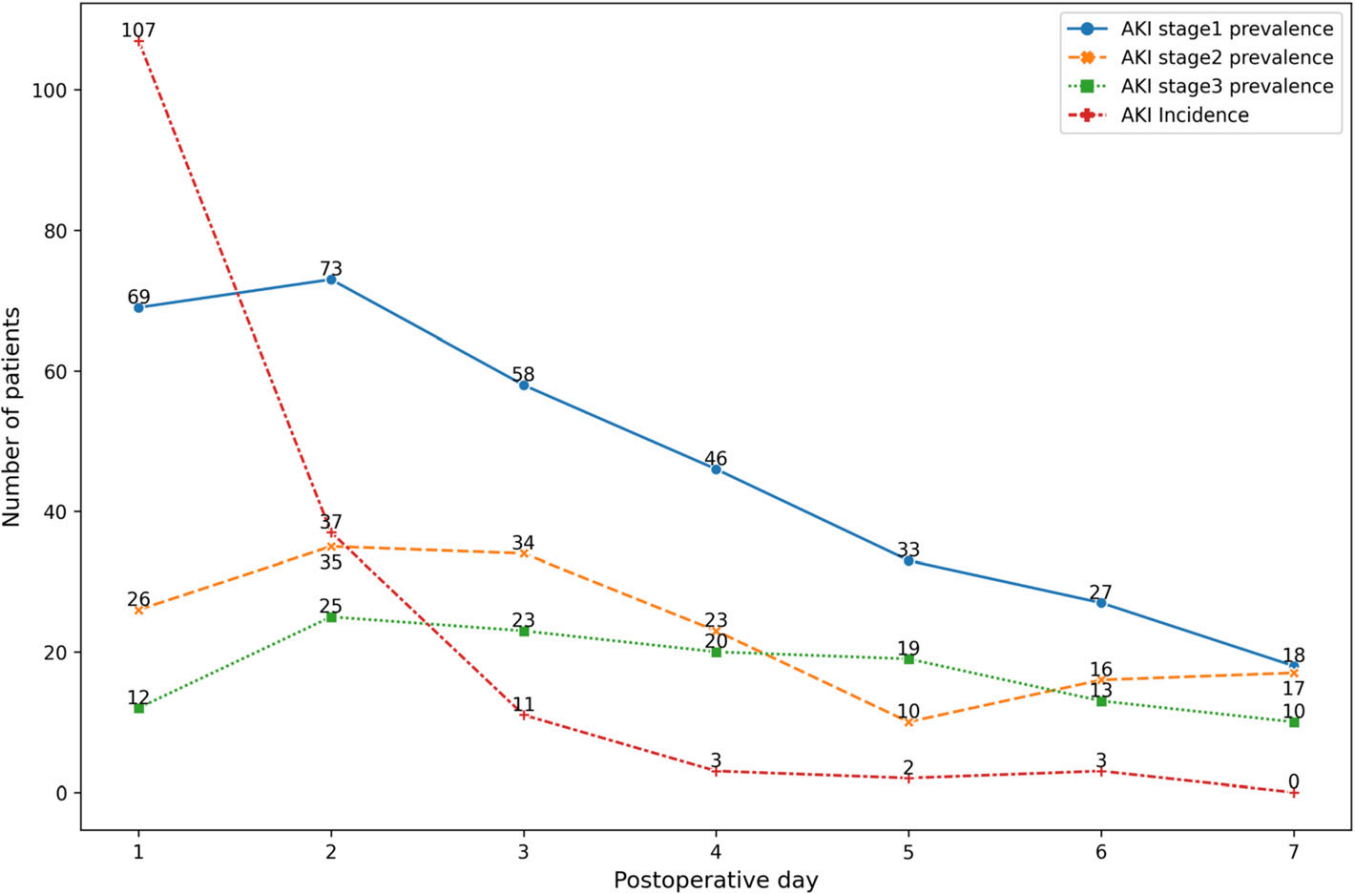
AKI incidence and prevalence during immediate postoperative time

Overall, 163 patients (66.8%) were subsequently diagnosed with AKI after OLT (Table 2). Approximately, 65.6% (107/163) of AKI occurred on the first postoperative day (Fig. 2). Among those patients, 76 (31.3%) were classified as stage 1, 52 (21.3%) as stage 2 and 35 (14.3%) as stage 3 acute kidney injuries. The incidence of severe AKI (stage 2/3) was 0.0, 28.7, 47.7 and 50.5% in the underweight group, normal weight group, overweight group and obese group, respectively.

**Table 2:** Incidence of AKI and additional postoperative outcomes after liver transplantation, Stratified by BMI

Classification	Total (*n* = 244)	Underweight (*n* = 8)	Normal weight (*n* = 136)	Overweight (*n* = 86)	Obese (*n* = 14)	*P* Value
No AKI	81 (33.2%)	5 (62.5%)	51 (37.5%)	22 (25.6%)	3 (21.4%)	0.060
Overall AKI	163 (66.8%)	3 (37.5%)	85 (62.5%)	64 (74.4%)	11 (78.6%)	0.038
AKI stage 1	76 (31.1%)	3 (37.5%)	46 (33.8%)	23 (26.7%)	4 (28.6%)	
AKI stage 2	52 (21.3%)	0 (0.0%)	26 (19.1%)	21 (24.4%)	5 (35.7%)	
AKI stage 3	35 (14.3%)	0 (0.0%)	13 (9.6%)	20 (23.2%)	2 (14.3%)	
Severe AKI (stage 2/3)	87 (35.6%)	0 (0.0%)	39 (28.7%)	41 (47.7%)	7 (50.0%)	0.002
RRT	12 (4.9%)	0 (0.0%)	3 (2.2%)	8 (9.3%)	1 (7.1%)	0.090
Postoperative hospitalization, median (IQR), day	26 (20–28)	27 (24–44)	25 (20–28)	26 (21–29)	28 (23–29)	0.582
Hospital mortality, No. (%)	7 (2.9%)	0 (0.0%)	4 (2.9%)	1 (1.2%)	2 (14.3%)	0.103

### Overweight and postoperative AKI

**Fig. 3:**
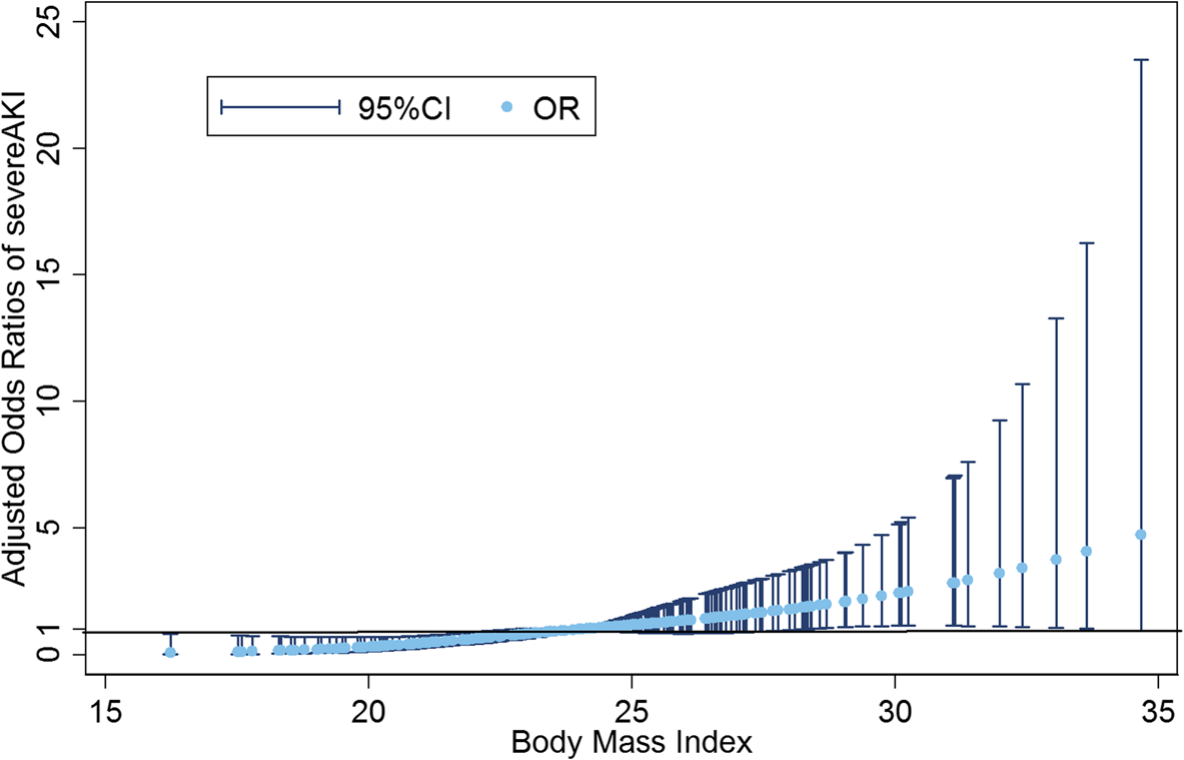
Non-linear relationship between BMI and AKI after OLT. Legend: Cubic spline analysis showed J-shaped association of BMI and postoperative severe AKI after adjusting for confounding factors

We used a restricted cubic spline to flexibly model and visualize the non-linear relationship. After adjusted for confounding factors, the association between BMI and AKI after OLT were J-shaped (Fig. 3). In the primary analyses (model 1), there were no statistically significant differences in postoperative AKI among different BMI categories. However, overweight was associated with a higher incidence of postoperative severe AKI compared with normal weight (crude OR: 2.266; 95% CI, 1.290–3.980; *P* = 0.004). This relationship between overweight and postoperative severe AKI remained robust (adjusted OR: 2.539; 95% CI, 1.389–4.642; *P* = 0.002) in the fully adjusted model (model 3). Furthermore, when compared with normal weight, patients with obese were at even higher risk of postoperative severe AKI (adjusted OR: 3.705; 95% CI, 1.108–12.388; *P* = 0.033), independent of traditional risk factors. (Table 3).

**Table 3:** Association of Overweight with Postoperative AKI

Outcome	Model 1		Model 2		Model 3	
	OR (95%CI)	*P* Value	OR (95%CI)	*P* Value	OR (95%CI)	*P* Value
AKI						
Normal weight	1 (reference)		1 (reference)		1 (reference)	
Underweight	0.360 (0.083–1.570)	0.174	0.240 (0.051–1.140)	0.073	0.219 (0.045–1.063)	0.059
Overweight	1.745 (0.962–3.168)	0.067	1.820 (0.985–3.363)	0.056	1.781 (0.962–3.297)	0.066
Obese	2.200 (0.586–8.260)	0.243	2.522 (0.633–10.052)	0.190	2.427 (0.608–9.684)	0.209
Severe AKI (stage 2/3)						
Normal weight	1 (reference)		1 (reference)		1 (reference)	
Underweight	–	–	–	–	–	–
Overweight	2.266 (1.290–3.980)	0.004	2.560 (1.401–4.678)	0.002	2.539 (1.389–4.642)	0.002
Obese	2.487 (0.818–7.559)	0.108	3.741 (1.119–12.510)	0.032	3.705 (1.108–12.388)	0.033

Note: - not available

Model 1 was unadjusted

Model 2 was adjusted for a priori defined variables based on literature research and clinical relevance (female gender, CTP score, MELD score, pre-existing CKD, pre-existing DM, preoperative Scr, requirement of vasopressors, postoperative peak AST, Tacrolimus)

Model 3 was adjusted for the same variables as model 2 and for potential confounding factors based on univariate analyses (female gender, preoperative Scr, preoperative lactic acid, intraoperative RBC transfusion)

### Overweight and other perioperative outcomes

There were no statistically significant differences in postoperative ventilation time, postoperative hospitalization, or hospital mortality among underweight, normal weight, overweight and obese groups (Table 2).

## Discussion

In this retrospective cohort study at our center, 163 (66.8%) patients who received OLT were diagnosed with postoperative AKI. Among these 163 patients, 87 (53.4%) developed severe AKI (stage 2/3) and 12 (7.4%) required RRT during postoperative hospitalization. Overweight were associated with an increased risk of postoperative severe AKI and this relationship was independent of known confounding factors.

Postoperative AKI is a common complication following OLT. In keeping with previous studies [1], the overall incidence of postoperative AKI and postoperative severe AKI at our center were 66.8 and 35.6%, respectively. The slightly higher incidence of AKI may be due to relatively lower diagnostic threshold of KDIGO criteria. This lends further support to the notion that it is time to pay close attention to postoperative AKI, as even transient AKI is associated with worse patients and graft outcomes [22].

Our results suggest that overweight is associated with an increased risk for severe AKI after OLT. A couple of explanations can be given for this association. First, while the pathophysiology of postoperative AKI is multifactorial and may have a complex interplay of each other, a major factor may be hypoperfusions of kidney and subsequent systemic and local processes that can occur during surgery [23]. Specifically, adequacy fluid resuscitation of overweight especially obese patients creates additional challenges since it is still uncertain whether the dose based on actual body weight or by formula-based weight adjustment [17], Overweight and obesity also make it more complicated and challenging to operative field exposure and certain surgical procedures such as clamping and unclamping the portal vein which is often followed by hemodynamic instability [4]. Next, as a component of metabolic syndrome, obesity is also a significant risk factor for cardiovascular disease, hypertension, DM, and CKD, which may provide a vulnerable physiological reserve to handle the stress hypoperfusion of kidney during surgery [24]. Then, overweight and obesity leads to glomerular hyperfiltration, augmented urinary albumin loss, and deterioration of renal function related to a focal segmental glomerulosclerosis. The obesity-associated ultrastructural and functional changes are collectively called obesity-related glomerulopathy [17]. Besides, obesity induced a chronic mild inflammatory condition with increased adipokines, and evidence has shown that adiponectin may have a pivotal role in the pathogenesis of acute renal ischemia/reperfusion injury [25, 26]. Moreover, patients with increasing BMI showed a rise in markers of oxidative stress and endothelial dysfunction, which are stronger predictors of postoperative AKI than inflammatory markers [27]. As such it is plausible that overweight results in an increased risk for postoperative severe AKI.

An animal study reported that obese swine receiving the surgery procedure presented with attenuated kidney dysfunction after cardiac surgery, Sleeman and colleagues proposed that chronic hyper-inflammatory status induced by pre-operative obesity had ‘pre-conditioning’ effects against ischemia/reperfusion injury as a possible explanation of the phenomenon [28]. In the study of Ju Yeon Park, no significant difference was found in occurrence of AKI after OLT between underweight and normal weight patients [29]. So far, systemic reviews aiming to understand the relationships between obesity and postoperative AKI have not been thoroughly performed in patients undergoing OLT. A previous study suggested that patients with BMI > 25 should get more attention even in patients with normal preoperative renal function [30]. Another study included recipient BMI as a predictor in their prediction model for AKI after liver transplantation [31]. In the study of Hilmi, weight > 100 kg was associated with the development of AKI within 72 h [5]. In addition, overweight has been studied in general surgery and other clinical settings showing similar outcomes [32]. Our results support these findings and our study is unique by stratifying patients by different BMI categories and the J-shaped association of BMI and severe AKI after OLT. Together these results suggest that overweight is a strong modifiable risk factor of severe AKI after OLT.

Obesity paradox has been an area of intensive investigation in diverse clinical settings over the past few years. It refers to the phenomenon that being overweight and moderately obese was actually associated with superior clinical outcomes (compared with patients with normal and low BMI) [33]. For example, In the study of Moon H, patients in the overweight groups showed a higher risk of postoperative AKI, but these patients had a more favorable prognosis in terms of overall mortality after coronary artery bypass grafting [32]. In another study that was based on patients in the ICU, an increased BMI was highly associated with the development of AKI, but with a decreased in-hospital mortality [34]. With respect to OLT, an recent meta-analysis concluded that BMI > 30 increased post-transplant complications, though length of hospital or ICU were not increased as compared to controls [14]. However, our results showed no relationship between overweight and other immediate postoperative outcomes. Future study is required with wider populations, better markers combined with animal studies to fully elucidate the physiological mechanism of obesity paradox and underlying obesity-related comorbidities. In addition, we will continue to explore the impact of BMI on progression to CKD and mortality in OLT recepients with AKI.

### Strengths and limitations

This study examines whether overweight is associated with the risk for postoperative AKI and other postoperative outcomes in patients receiving OLT. We were able to examine a cohort of patients who had a OLT at our center and we controlled for various confounders, including patient and surgery factors. In addition, we used restricted cubic spline to analysize the non-linear relationship of BMI and severe AKI after OLT, and it turned out to be J-shaped.

This study has several limitations, including but not limited to its retrospective nature. The occurrence of AKI was identified using SCr. Although it is the most widely used marker for renal function in clinical practice, it is likely that SCr overestimates preoperative renal function and delayed diagnosis and underestimate the severity of postoperative AKI, as patients awaiting OLT tend to have a reduced creatinine production compared with healthy subjects and fluid accumulation might mask the increase in SCr [3]. We mitigated this limitation by defining the baseline SCr from most recent SCr measurement made 3 to 90 days prior to operation. The incidence of postoperative AKI in our cohort (66.8%) is consistent with previous reports. A further limitation was our definition of overweight, defined as BMI ≥ 25 kg/m^2^. BMI per se has limitations since it cannot represent body composition especially in scenarios of sarcopenic obesity and oedema or ascites [14]. We did not take into account the influence of a correction of BMI according to the volume of ascites because ascites drained at the time of OLT did not differ significantly between groups in our study. Also, potential unmeasured confounding remains from variables not measured in our study (eg, donor factors, use of angiotensin-converting enzyme inhibitors, reperfusion syndrome). Future studies should clarify whether these variables are related to the occurrence and severity of AKI after OLT.

## Conclusions

Overweight is associated with an increased risk of postoperative severe AKI among patients receiving OLT. Overweight is an independent risk factor of postoperative severe AKI among patients receiving OLT after adjusting for confounding factors. There were no statistically significant differences in postoperative outcomes among different BMI categories.

## Data Availability

The datasets used and analysed during the current study are available from the corresponding author on reasonable request.

## Abbreviations

AKI: Acute kidney injury
OLT: Orthotopic liver transplantation
RRT: Renal replacement therapy
IRI: Hepatic ischaemia reperfusion injury
BMI: Body mass index
STROBE: Strengthening the Reporting of Observational Studies in Epidemiology
KIDGO: Kidney Disease, Improving Global Outcome
SCr: Serum creatinine
DM: Diabetes mellitus
CKD: Chronic kidney disease
HBV: Hepatitis B virus
HCV: Hepatitis C virus
MELD: Model for End-Stage Liver Disease
RBC: Red blood cell
AST: Aspartate transaminase
SD: Standard deviation
IQR: Interquartile range
OR: Odds ratio
CI: Confidence interval
ICU: Intensive care unit

## References

[1] Thongprayoon C, Kaewput W, Thamcharoen N, Bathini T, Watthanasuntorn K, Lertjitbanjong P, Sharma K, Salim SA, Ungprasert P, Wijarnpreecha K (2019). Incidence and impact of acute kidney injury after liver transplantation: a meta-analysis. J Clin Med.

[2] Zongyi Y, Baifeng L, Funian Z, Hao L, Xin W (2017). Risk factors of acute kidney injury after orthotopic liver transplantation in China. Sci Rep.

[3] de Haan JE, Hoorn EJ, de Geus HRH (2017). Acute kidney injury after liver transplantation: recent insights and future perspectives. Best Pract Res Clin Gastroenterol.

[4] Durand F, Francoz C, Asrani SK, Khemichian S, Pham TA, Sung RS, Genyk YS, Nadim MK (2018). Acute kidney injury after liver transplantation. Transplantation.

[5] Hilmi IA, Damian D, Al-Khafaji A, Planinsic R, Boucek C, Sakai T, Chang CCH, Kellum JA (2015). Acute kidney injury following orthotopic liver transplantation: incidence, risk factors, and effects on patient and graft outcomes. Br J Anaesth.

[6] Schlegel A, Linecker M, Kron P, Györi G, De Oliveira ML, Müllhaupt B, Clavien P-A, Dutkowski P. Risk assessment in high and low MELD liver transplantation. Am J Transplant. 2017;17(4):1050–63. 10.1111/ajt.14065 27676319

[7] Cabezuelo JB, Ramírez P, Ríos A, Acosta F, Torres D, Sansano T, Pons JA, Bru M, Montoya M, Bueno FS (2006). Risk factors of acute renal failure after liver transplantation. Kidney Int.

[8] Karapanagiotou A, Kydona C, Dimitriadis C, Sgourou K, Giasnetsova T, Fouzas I, Imvrios G, Gritsi-Gerogianni N (2012). Acute kidney injury after orthotopic liver transplantation. Transplant Proc.

[9] Lee SK, Park JB, Kim S-J, Choi GS, Kim DJ, CHD K, Lee SK, Joh JW. Early Postoperative Renal Dysfunction in the Adult Living Donor Liver Transplantation. Transplant Proc. 2007;39(5):1517–9. 10.1016/j.transproceed.2006.11.018 17580177

[10] Leithead JA, Armstrong MJ, Corbett C, Andrew M, Kothari C, Gunson BK, Muiesan P, Ferguson JW (2013). Hepatic ischemia reperfusion injury is associated with acute kidney injury following donation after brain death liver transplantation. Transpl Int.

[11] Jochmans I, Meurisse N, Neyrinck A, Verhaegen M, Monbaliu D, Pirenne J (2017). Hepatic ischemia/reperfusion injury associates with acute kidney injury in liver transplantation: prospective cohort study. Liver Transpl.

[12] Utsumi M, Umeda Y, Sadamori H, Nagasaka T, Takaki A, Matsuda H, Shinoura S, Yoshida R, Nobuoka D, Satoh D (2013). Risk factors for acute renal injury in living donor liver transplantation: evaluation of the RIFLE criteria. Transpl Int.

[13] Park MH, Shim HS, Kim WH, Kim HJ, Kim DJ, Lee SH, Kim CS, Gwak MS, Kim GS. Clinical risk scoring models for prediction of acute kidney injury after living donor liver transplantation: A retrospective observational study. PLoS One. 2015;10(8):e0136230. 10.1371/journal.pone.0136230 PMC4547769 26302370

[14] Leandro G, Viggiani MT, Losurdo G, Barone M, Principi M, Di Leo A (2017). Systematic review with meta-analysis: post-operative complications and mortality risk in liver transplant candidates with obesity. Aliment Pharmacol Ther.

[15] Tjeertes EEKM (2015). Obesity – a risk factor for postoperative complications in general surgery?. BMC Anesthesiol.

[16] Hafner S, Hillenbrand A, Knippschild U, Radermacher P. The obesity paradox and acute kidney injury: beneficial effects of hyper-inflammation? Crit Care. 2013;17(6):1023. 10.1186/cc13152 PMC4059416 24326122

[17] Suneja M, Kumar AB (2014). Obesity and perioperative acute kidney injury: A focused review. J Crit Care.

[18] Ersoy Z, Ozdemirkan A, Pirat A, Torgay A, Arslan G, Haberal M (2015). Perioperative characteristics of siblings undergoing liver or kidney transplant. Exp Clin Transplant.

[19] Elm EV, Altman DG, Egger M, Pocock SJ, Gotzsche PC, Vandenbroucke JP (2007). Strengthening the reporting of observational studies in epidemiology (STROBE) statement: guidelines for reporting observational studies. Br Med J.

[20] Rosique F, Cabezuelo JB, Ferreras D, González-Sánchez MR, Ros J, Pons JA, Cascales-Campos PA, Sánchez-Bueno F, Robles R, Ramírez P (2018). Long-term survival and evolution of the kidney function for liver transplant patients who required postoperative dialysis. Transplant Proc.

[21] Disease K (2012). Kidney Disease: improving global outcomes (KDIGO) acute kidney injury work group. Kidney Int.

[22] De Virgilio C, Kim DY (2016). Transient acute kidney injury in the postoperative period. Jama Surg.

[23] Meersch M, Schmidt C, Zarbock A (2015). Perioperative acute kidney injury. Anesth Analg.

[24] Kwakernaak AJ, Toering TJ, Navis G (2015). Body mass index and body fat distribution as renal risk factors.

[25] Oh J, Rabb H (2013). Adiponectin: an enlarging role in acute kidney injury. Kidney Int.

[26] Jin X, Chen J, Hu Z, Chan L, Wang Y (2013). Genetic deficiency of adiponectin protects against acute kidney injury. Kidney Int.

[27] KMP (2007). Visceral and subcutaneous adipose tissue volumes are cross-Sectionally related to markers of inflammation and oxidative stress: the Framingham heart study. Circulation.

[28] Sleeman P, Patel NN, Lin H, Walkden GJ, Murphy GJ (2013). High fat feeding promotes obesity and renal inflammation and protects against post cardiopulmonary bypass acute kidney injury in swine. Crit Care.

[29] Park JY, Park J-H, Lee SS, Ri H-S, Kim H-J, Choi YM, Choi YJ, Yoon J-U (2017). The Association of Preoperative Body Mass Index with acute kidney injury in liver transplantation recipients: a retrospective study. Korean J Crit Care Med.

[30] Tan L, Yang Y, Ma G, Zhu T, Yang J, Liu H, Zhang W (2019). Early acute kidney injury after liver transplantation in patients with normal preoperative renal function. Clin Res Hepatol Gastroenterol.

[31] Kalisvaart M, Schlegel A, Umbro I, de Haan JE, Polak WG, Ijzermans JN, Mirza DF, Perera MTP, Isaac JR, Ferguson J (2019). The AKI prediction score: a new prediction model for acute kidney injury after liver transplantation. HPB (Oxford).

[32] Hongran M, Yeonhee L, Sejoong K, Dong K, Ho J (2017). Differential signature of obesity in the relationship with acute kidney injury and mortality after coronary artery bypass grafting. Korean J Crit Care Med.

[33] Amundson DE, Djurkovic S, Matwiyoff GN. The obesity paradox. Crit Care Clin. 2010;26(4):583–96. 10.1016/j.ccc.2010.06.004 20970043

[34] Danziger J, Chen KP, Lee J, Feng M, Mukamal KJ (2015). Obesity, acute kidney injury, and mortality in critical illness. Crit Care Med.

